# Tracheotomy for Croup

**Published:** 1880-05

**Authors:** 


### TRACHEOTOMY FOR CROUP.

THE following conclusions are reached in an exhaustive paper on the subject in *Gaillard's Medical Journal*, January, 1880:

1. That tracheotomy is per se almost devoid of danger.

2. That fatal hemorrhage should almost never occur, and care with coolness will nearly always prevent opnœa from intracheal bleeding.

3. That age offers no contra-indications, although the average of success is less in early infancy and adult life.

4. That early operative interference, whenever the paroxysms of dyspnœa become at all lengthened, is demanded, since delay only adds to the suffering of the patient, and materially lessens the chances of recovery.

5. That the after-attention is of prime importance; careful attention of the wound, proper treatment of the disease, and proper nursing with fair hygienic surroundings, being the essentials to a successful issue.

---

WINE is the work of God; drunkenness is the work of the devil.—*St. John Chrysostom.*

